# TGF-β1 induces formation of TSG-6-enriched extracellular vesicles in fibroblasts which can prevent myofibroblast transformation by modulating Erk1/2 phosphorylation

**DOI:** 10.1038/s41598-024-62123-x

**Published:** 2024-05-29

**Authors:** Marcus M. Ilg, Stephen A. Bustin, David J. Ralph, Selim Cellek

**Affiliations:** 1 Medical Technology Research Centre, HEMS, SoAH, Anglia Ruskin University, Chelmsford, CM1 1SQ UK; 2 Urology Department, University College London, London, UK

**Keywords:** Mechanisms of disease, Cell biology, Cell signalling, Extracellular signalling molecules

## Abstract

Extracellular vesicles have emerged as important mediators of cell-to-cell communication in the pathophysiology of fibrotic diseases. One such disease is Peyronie’s disease (PD), a fibrotic disorder of the penis caused by uncontrolled transformation of resident fibroblasts to alpha-smooth muscle actin positive myofibroblasts. These cells produce large amounts of extracellular matrix, leading to formation of a plaque in the penile tunica albuginea (TA), causing pain, penile curvature, and erectile dysfunction. We have used primary fibroblasts derived from the TA of PD patients to explore the role of transforming growth factor beta 1 (TGF-β1), a key signalling factor in this process. TGF-β1 treatment elicited a range of responses from the myofibroblasts: (i) they secreted extracellular vesicles (EVs) that were more numerous and differed in size and shape from those secreted by fibroblasts, (ii) these EVs prevented TGF-β1-induced transformation of fibroblasts in a manner that was dependent on vesicle uptake and (iii) they prevented phosphorylation of Erk1/2, a critical component in modulating fibrogenic phenotypic responses, but did not affect TGF-β1-induced Smad-signalling. We posit that this effect could be linked to enrichment of TSG-6 in myofibroblast-derived EVs. The ability of myofibroblast-derived vesicles to prevent further myofibroblast transformation may establish them as part of an anti-fibrotic negative feedback loop, with potential to be exploited for future therapeutic approaches.

## Introduction

Fibrosis is a consequence of dysregulated wound healing and can affect any organ in the body^1^. Its main characteristic is the excessive accumulation of extracellular matrix (ECM) components such as fibronectin or collagens, which are produced in response to an injury. Although fibrosis contributes to 45% of all-cause mortality world-wide, treatment options remain limited^2^. Peyronie’s disease (PD) is a fibrotic disorder of the penile tunica albuginea (TA) that affects up to 9% of the male population^3^. The formation of a fibrous plaque can lead to pain, penile curvature and erectile dysfunction^4^, resulting in significant physical and mental distress. This, together with uncertainty surrounding the exact aetiology and pathophysiology of PD and the fact that there is no effective medical treatment^5,6^, underlines the importance of investigating fibrosis in PD.

A key aspect of PD is the transformation of resident fibroblasts to alpha-smooth muscle actin (α-SMA) -positive, ECM producing and contractile myofibroblasts^7,8^. These cells are responsible for the promotion of wound contraction, collagen turnover and ECM remodelling^1,9^. Upon completion of the normal wound healing response, myofibroblasts undergo apoptosis^10^, resulting in only a transient increase in ECM production with restoration of functional tissue structure. However, in fibrosis the myofibroblasts become apoptosis-resistant, which leads to excessive accumulation of ECM where synthesis rate exceeds degradation rate^11^. This can lead to disrupted tissue architecture and ultimately organ failure^12^.

Local fibroblasts demonstrate this phenotypic plasticity upon tissue injury, particularly as a response to cytokines such as TGF-β1, which is the most studied inducer of myofibroblast transformation^13,14^. TGF-β1 is secreted in its latent form and is activated through cooperation from proteases, integrins, and specialised extracellular matrix molecules. It exerts its effects on fibroblast differentiation by activating intracellular signalling pathways that regulate the expression of genes involved in cell fate determination and extracellular matrix production^15^. Specifically, binding of TGF-βs to the constitutively active TβRII recruits and transphosphorylates the type I receptor kinases, followed by phosphorylation of the receptor-activated Smads. These form complexes with Smad4 and translocate to the nucleus, where they modulate gene transcription^16^. In addition to activation of this canonical cascade, TGF-ß can signal via non-canonical pathways, including MAPK family pathways such as ERK, JNK, MAPK^17^. TGF-β1 has also been shown to change cellular metabolism in fibroblasts, leading to increased production of chemical building blocks such as amino acids and fatty acids^18–20^.

Extracellular vesicles (EVs) are lipid bound vesicles which are released by virtually all cell types^21^. Their importance on cellular communication is well established^22^ and they can be divided into three main subtypes, microvesicles, exosomes, and apoptotic bodies, based upon their biogenesis^23–25^. Their subpopulations can differ in size (40–1,000 nm), morphology, composition and cargo, carrying distinct sets of nucleic acids, proteins or metabolites^26–28^. They can contain miRNAs, mRNAs, lncRNAs, or circular RNAs^29^. Whilst they can be messengers of bioactive cargo, their signalling capacity can also depend on the components on the membrane, activating receptors without releasing any cargo^30^. Despite their critical role in intercellular communication, their importance in fibrotic disorders has only recently been acknowledged and is still understudied in PD^31^.

This study aimed to investigate the effects of TGF-β1 on the form and function of EVs in primary human fibroblasts derived from patients with PD.

## Results

### Analysis of extracellular vesicles

**Figure 1:**
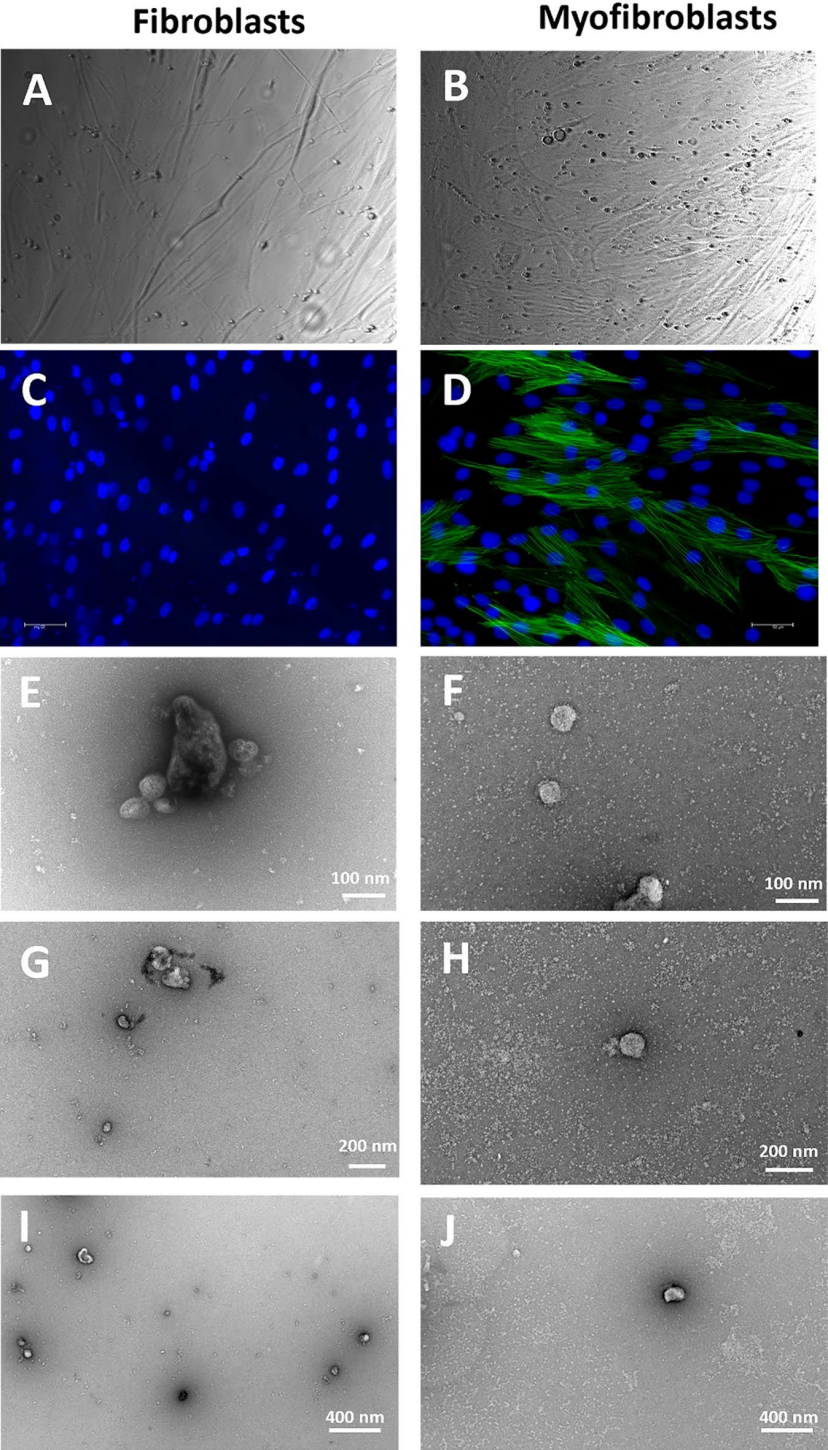
Visual confirmation of fibroblast and myofibroblast status and TEM images of EVs. Cell phenotype was confirmed via phase contrast images and ICC staining. Fibroblast phenotype shown in (**A**,**C**) Myofibroblast phenotype shown in (**B**,**D**) as shown by changed morphology and expression of a-SMA stress fibres (green). Nuclear stain in blue (DAPI). Negative staining transmission electron microscopy images at 100 nm, 200 nm, and 400 nm of fibroblast-derived EVs (**E**,**G**,**I**) and myofibroblast-derived EVs (**F**,**H**,**J**).

The vesicle isolation protocol used to test the hypothesis that treatment with TGF-β1 results in altered EV production is summarized in Supplementary Fig. 1. Knockout Serum Replacement (KOSR) containing serum-free media was used to avoid any contaminating EVs originating from FCS. KOSR is a defined serum replacement^32^ and has been previously used for isolating EVs from human pluripotent stem cells^33^. Before isolation, myofibroblast transformation was confirmed visually (Fig. 1A and B) and also by expression of α-SMA using ICC (Fig. 1C and D). Fibroblasts are thinner and more spindle shaped compared to myofibroblasts, whilst the cell numbers were relatively similar (4.2 × 10^6^ total cells for fibroblasts, 3.9 × 10^6^ total cells for myofibroblasts), and viability was above MISEV guidelines (> 98 ± 1% for both fibroblasts and myofibroblasts)^24^ (Fig. 1). Two vesicle groups were obtained, EVs derived from fibroblasts (fibroblast EVs) and EVs derived from myofibroblasts which were transformed from fibroblasts using TGF-β1 (myofibroblast EVs). Vesicles were subjected to TEM and images at different magnifications of 100 nm, 200 nm and 400 nm are displayed for fibroblast EVs (Fig. 1E,G,I) and myofibroblast EVs (Fig. 1F,H,J), respectively. The images show representative morphology of the vesicles which were mostly round and there were EVs of multiple sizes in both groups.

**Figure 2:**
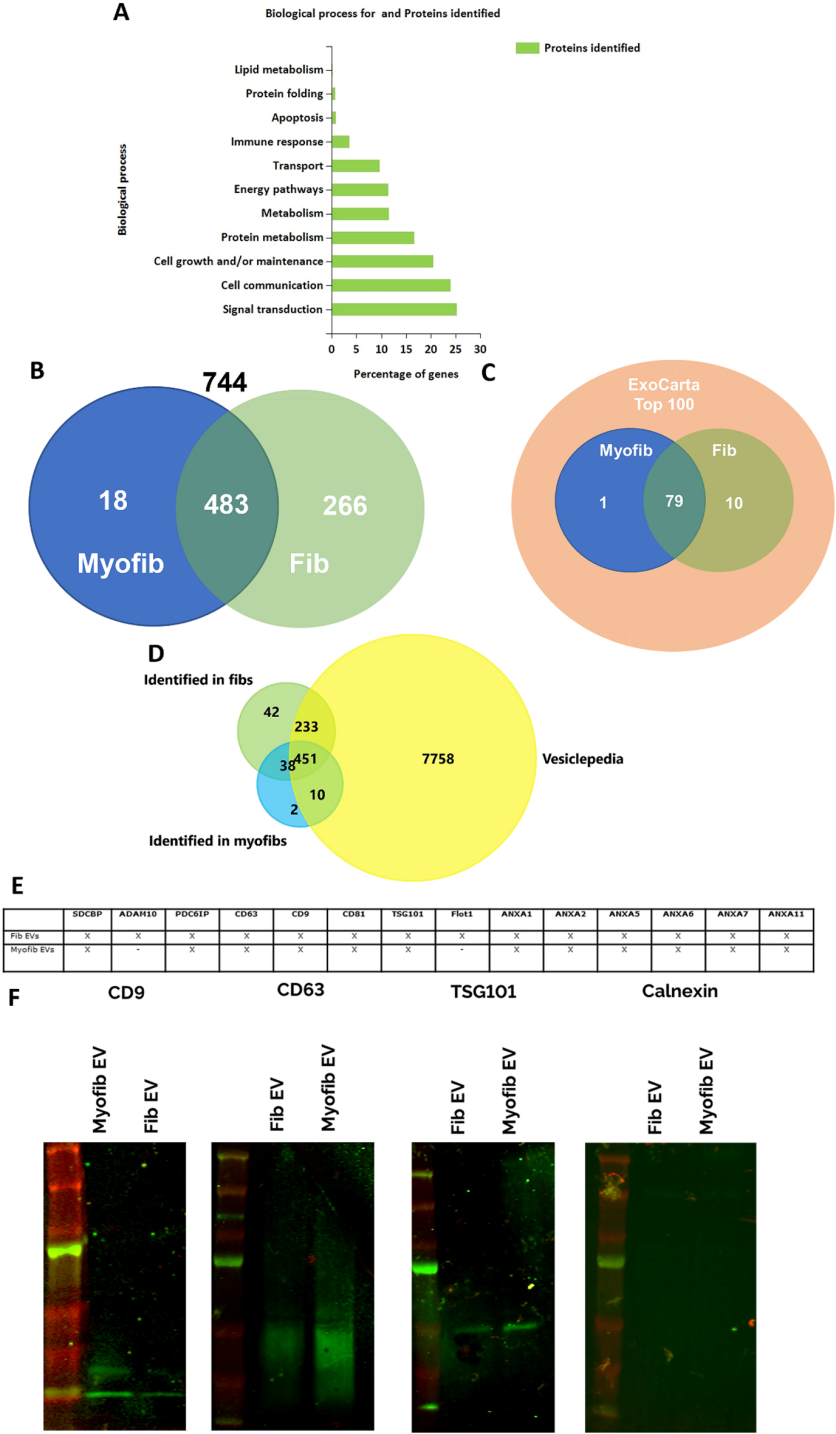
Protein-based characterisation of EVs. EVs produced by fibroblasts and myofibroblasts were subjected to proteomic and Western blot analysis. (**A**) Enrichment analysis for biological processes in all proteins identified in the samples. Graph generated using FunRich. (**B**) Venn diagram of proteins identified in fibroblast and myofibroblast EVs (total of 744). (**C**) Venn diagram of proteins identified in fibroblast and myofibroblast EVs when compared to ExoCarta top 100 frequently identified proteins. (**D**) Venn diagram of proteins identified in fibroblast and myofibroblast EVs when compared to Vesiclepedia database. (**E**) Frequently used markers to confirm EV status and their expression in fibroblast and myofibroblast EVs. X delineates expression,—no expression. (**F**) Western blot confirmation of some of the markers shown in E. Odyssey image outputs for CD9, CD63, TSG101, and calnexin, respectively. Note presence of green bands for CD9, CD63, and TSG101, suggesting expression of the markers in both, fibroblast and myofibroblast EVs. Membranes were cut before incubation with primary antibody. Full membranes shown in Supplementary Material.

Using proteomics analysis, a total of 744 proteins could be identified in fibroblast and myofibroblast EVs. Enrichment analysis revealed the main biological processes for the proteins identified (with the highest percentages attributed to signal transduction and cell communication), as seen in Fig. 2A. 483 of the 744 proteins were found in both groups while 18 were found only in myofibroblasts and 266 only in fibroblasts, as shown in the Venn diagram in Fig. 2B. To characterise the EVs, the data were compared to the ExoCarta Top 100, which includes proteins that are frequently found in exosomes. The myofibroblast EVs displayed 80 of the top 100 (1 of which was not found in the fibroblast EVs), whilst the fibroblast EVs showed 89 of the top 100 (Fig. 2C). In addition, comparison with the Vesiclepedia database showed that most of the proteins identified in fibroblast and myofibroblast EVs have been described previously (Fig. 2D), with both unique and shared exceptions for both fibroblast and myofibroblast EVs. Figure 2E shows 14 common EV markers^34^ and their expression in both fibroblast and myofibroblast EVs. Whilst fibroblast EVs are positive for all 14 markers, myofibroblast EVs did not express ADAM10 and Flot1. To further confirm the above marker expression, the vesicles were subjected to Western blot analysis. This confirmed that TSG101 (classical exosome marker) and the tetraspanin markers CD9 and CD63 were indeed present in the vesicles, with tetraspanins enriched in myofibroblasts. Additionally, the absence of endoplasmic reticulum protein calnexin was confirmed in both groups (Fig. 2F).

**Figure 3:**
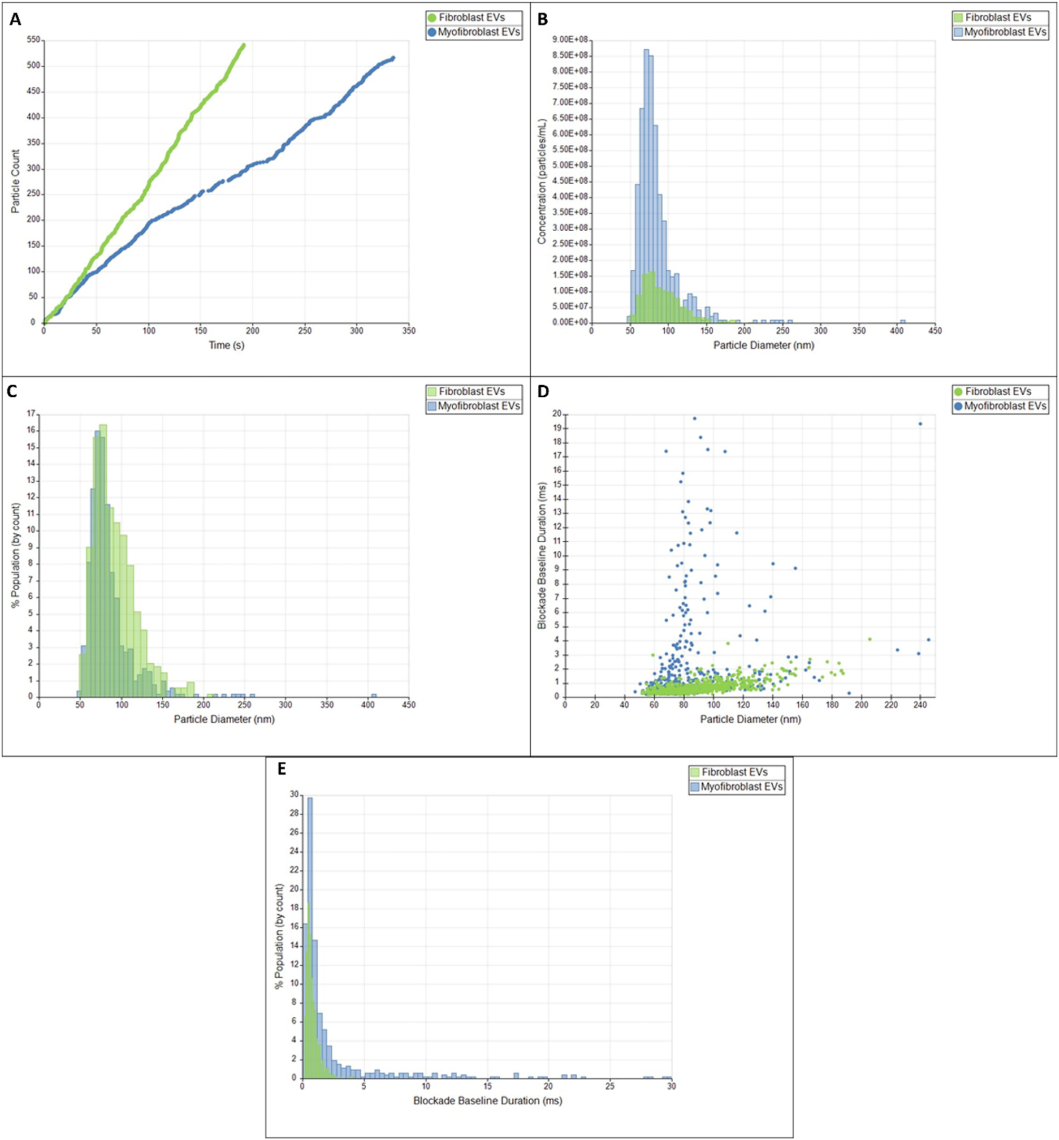
TRPS analysis of EVs produced by fibroblasts and myofibroblasts. Analysis of fibroblast EVs (green) and myofibroblast EVs (blue) using qNano Gold. Particle rate plot shows linearity for both samples (**A**). Particle diameter vs concentration/mL is shown in (**B**), showing that myofibroblasts produce more EVs overall. Particle diameter vs percentage population is plotted in (**C**) showing that fibroblasts produce more larger particles. Particle diameter is plotted vs blockage duration in (**D**) showing that myofibroblasts produce more particles with a longer blockage duration. Blockage duration is plotted vs percentage population in (**E**) showing that myofibroblasts produce more particles with higher blockage duration, whereas fibroblast EVs seem more uniform in shape. All images are representative images of one experiment. Experiments were repeated three times to obtain averages.

TRPS analysis revealed that the number, size and shape the vesicles particles produced was affected by treatment with TGF-β1 (Fig. 3). Linearity of the measurement (particle count over time) acted as quality control as seen in Fig. 3A. TGF-β1-treated cells produced 4.72 ± 0.8 × 10^9^ particles/mL, whilst untreated fibroblasts produced 0.72 ± 0.08 × 10^9^ particles/mL (p = 0.013, N = 5). Further, the particles produced by myofibroblasts were smaller (mean diameter 72.8 ± 4 nm compared to those produced by fibroblasts (mean diameter 90.4 ± 1 nm) as seen in Fig. 3B (p = 0.004, n = 5). Untreated cells showed a larger percentage population of particles > 80 nm compared to TGF-ß1-treated cells, which showed the highest concentration of particles at 70 nm (Fig. 3C). TRPS allows to make assumptions on the shape of particles based on how long it takes them to traverse the Nanopore (blocking duration). There were differences in shape between the particles, with the TGF-ß1-treated group showing more small but elongated particles, based on the blocking duration measured by TRPS (Fig. 3D and E) Further, myofibroblasts produced more EVs that had a longer blocking duration compared to fibroblast EVs (3.91 ± 0.29 ms vs. 0.77 ± 0.11 ms, p = 0.0006). No particles above 240 nm were detected in either group, classifying the particles investigated in this study mostly as small EVs (sEVs) (< 200 nm in diameter), which are traditionally enriched for exosomes^35^.

### Effect of vesicles on myofibroblast marker expression and role of vesicle uptake

**Figure 4:**
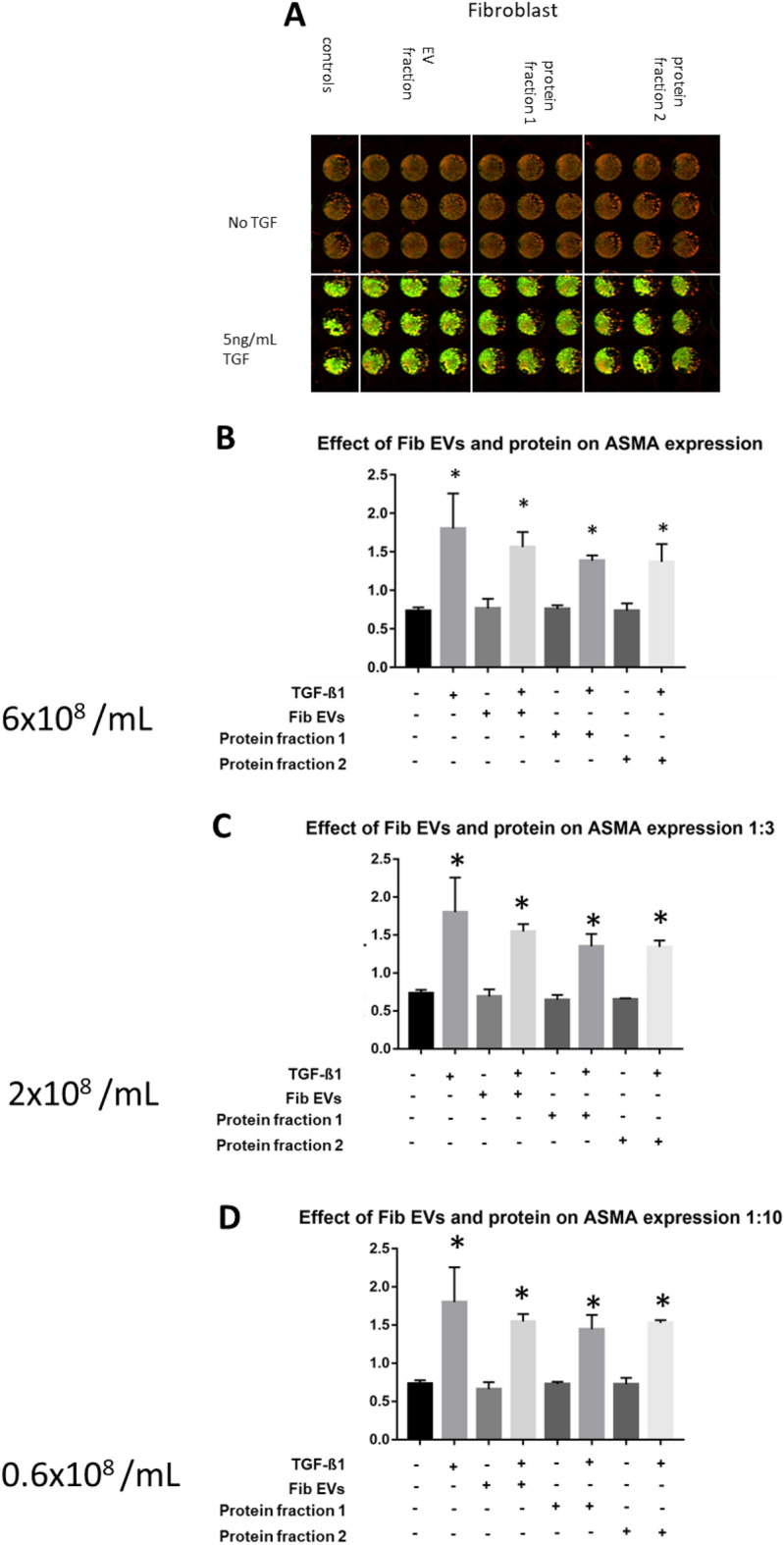
Effect of fibroblast vesicles and soluble protein fractions on myofibroblast transformation. Fibroblasts were treated with various concentrations of fibroblast EVs or soluble protein fractions in presence or absence of 5 ng/mL TGF-β1 for 72 h after which myofibroblast transformation was quantified using ICE for a-SMA. (**A**) Odyssey image output showing plate layout and results with red indicating nuclear staining and green indicating a-SMA expression. (**B**) Quantification of highest concentration of EVs (6 × 10^8^ /mL) and soluble protein + /- TGF-β1. No effect shown for any EV or protein fraction. (**C**) Quantification of middle concentration of EVs (2 × 10^8^ /mL) and soluble protein +/− TGF-β1. No effect shown for any EV or protein fraction. (**D**) Quantification of lowest concentration of EVs (0.6 × 10^8^ /mL) and soluble protein +/− TGF-β1. Data shown as ratio a-SMA expression/DRAQ5 staining. No effect shown for any EV or protein fraction. *p < 0.05 vs. untreated cells. N = 3.

**Figure 5:**
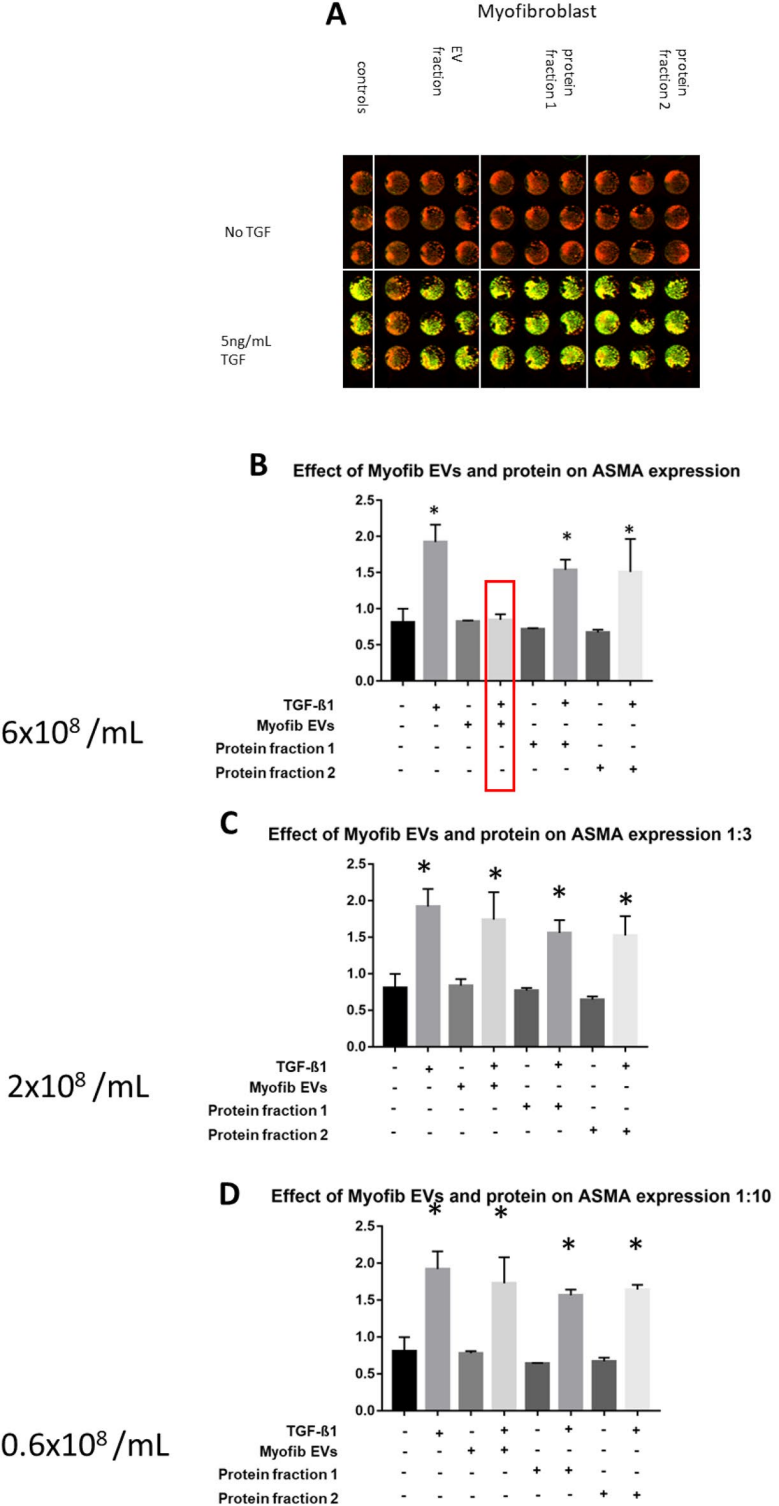
Effect of myofibroblast vesicles and soluble protein fractions on myofibroblast transformation. Fibroblasts were treated with various concentrations of myofibroblast EVs or soluble protein fractions in presence or absence of 5 ng/mL TGF-β1 for 72 h after which myofibroblast transformation was quantified using ICE for a-SMA. (**A**) Odyssey image output showing plate layout and results with red indicating nuclear staining and green indicating a-SMA expression. (**B**) Quantification of highest concentration of EVs (6 × 10^8^ /mL) and soluble protein +/− TGF-β1. Myofibroblast EVs show no significant difference to untreated cells despite presence of TGF-β1 (red box). (**C**) Quantification of middle concentration of EVs (2 × 10^8^ /mL) and soluble protein +/− TGF-β1. No effect shown for any EV or protein fraction. (**D**) Quantification of lowest concentration of EVs (0.6 × 10^8^ /mL) and soluble protein +/− TGF-β1. Data shown as ratio a-SMA expression/DRAQ5 staining. No effect shown for any EV or protein fraction. *p < 0.05 vs. untreated cells. N = 3.

To investigate whether the isolated vesicles evoke any biological effect, the expression of α-SMA was measured in unstimulated fibroblasts exposed to isolated vesicles and co-isolated protein fractions in the absence or presence of TGF-β1. The following concentrations were tested: 6 × 10^8^/mL, 2 × 10^8^/mL, 0.6 × 10^8^/mL. Protein fractions were tested so as to exclude the possibility of soluble proteins causing any observed effect. α-SMA expression was quantified after a 72 h incubation using ICE (Fig. 4 A–D). EVs obtained from fibroblasts did not elicit any effect on α-SMA expression at any concentration either alone or with TGF-β1. In contrast, EVs obtained from myofibroblasts prevented the TGF-β1-induced alpha-smooth muscle actin expression in fibroblasts at the highest concentration tested (p < 0.05) (Fig. 5B–D). This effect was limited to the EVs, while neither the early eluting, nor the late eluting protein fractions elicited such effect (Fig. 4B–D). They did not change the a-SMA expression in fibroblasts not exposed to TGF-ß1.

To exclude the possibility of the media components, rather than myofibroblast EVs, affecting myofibroblast transformation, blank KOSR media was subjected to the same EV and soluble protein fraction isolation method as the myofibroblast conditioned media. There was no anti-fibrotic effect in any of the fractions when combined with TGF-β1 Figure Supplementary Fig. 2.

To further substantiate the biological effect being myofibroblast-specific, conditioned media from rhabdomyosarcoma cells (RD) was generated and subjected to the same isolation method as the fibroblast/myofibroblast samples. Rhabdomyosarcoma cells originate from skeletal muscle and express desmin^36^, and they do not possess the other characteristics of fibroblasts or myofibroblasts. None of the fractions isolated from RD cells affected myofibroblast transformation significantly (Supplementary Fig. 3).

**Figure 6:**
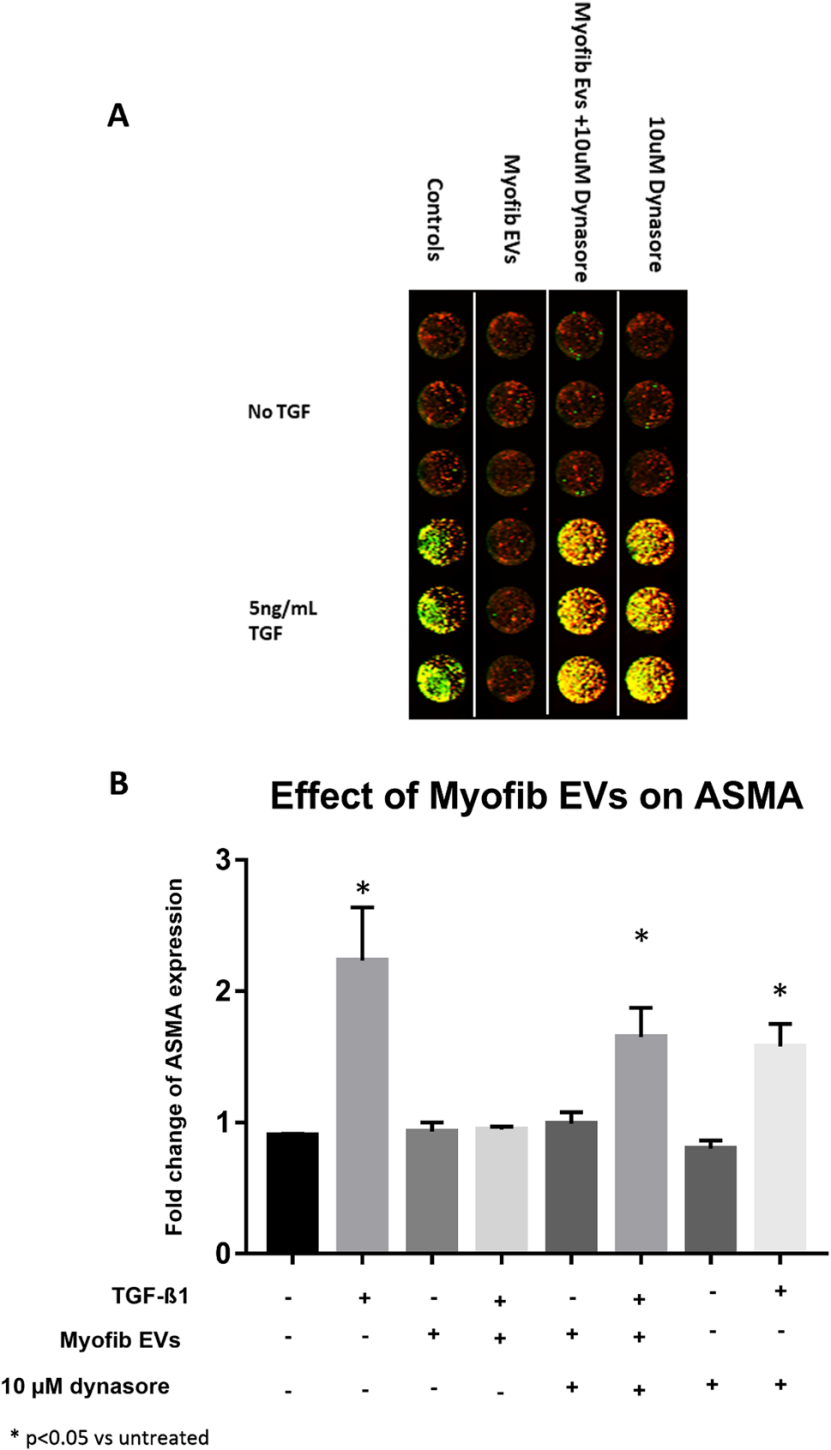
Effect of uptake inhibitor on myofibroblast vesicle effect. Fibroblasts were treated with myofibroblast EVs (6 × 10^8^ /mL) in presence or absence of 5 ng/mL TGF-β1 and/or uptake inhibitor dynasore (10 μM) for 72 h after which myofibroblast transformation was quantified using ICE for a-SMA. (**A**) Odyssey image output showing plate layout and results with red indicating nuclear staining and green indicating a-SMA expression. (**B**) Quantification of effect of vesicles on a-SMA expression. Myofibroblast eVs could prevent a-SMA expression which was inhibited by presence of dynasore. *p < 0.05 vs untreated cells. N = 3.

The dependence of this biological effect on vesicle uptake was further confirmed by our finding that the addition of dynasore (10 μM) (cell-permeable dynamin inhibitor that blocks endocytosis; Ref.^37^) inhibited the prevention of TGF-β1-induced α-SMA expression by myofibroblast-derived EVs (Fig. 6).

### Effect of myofibroblast-derived vesicles on signalling cascades downstream of TGF-β1

The possible effects of myofibroblast-derived EVs on signalling cascades downstream of TGF-β1 was investigated in both canonical and non-canonical pathways. The best timepoint for measurement was determined by performing a TGF-β1 time course for pSmad2/3 and pErk1/2, two of the main effectors of TGF-β1 signalling.

**Figure 7:**
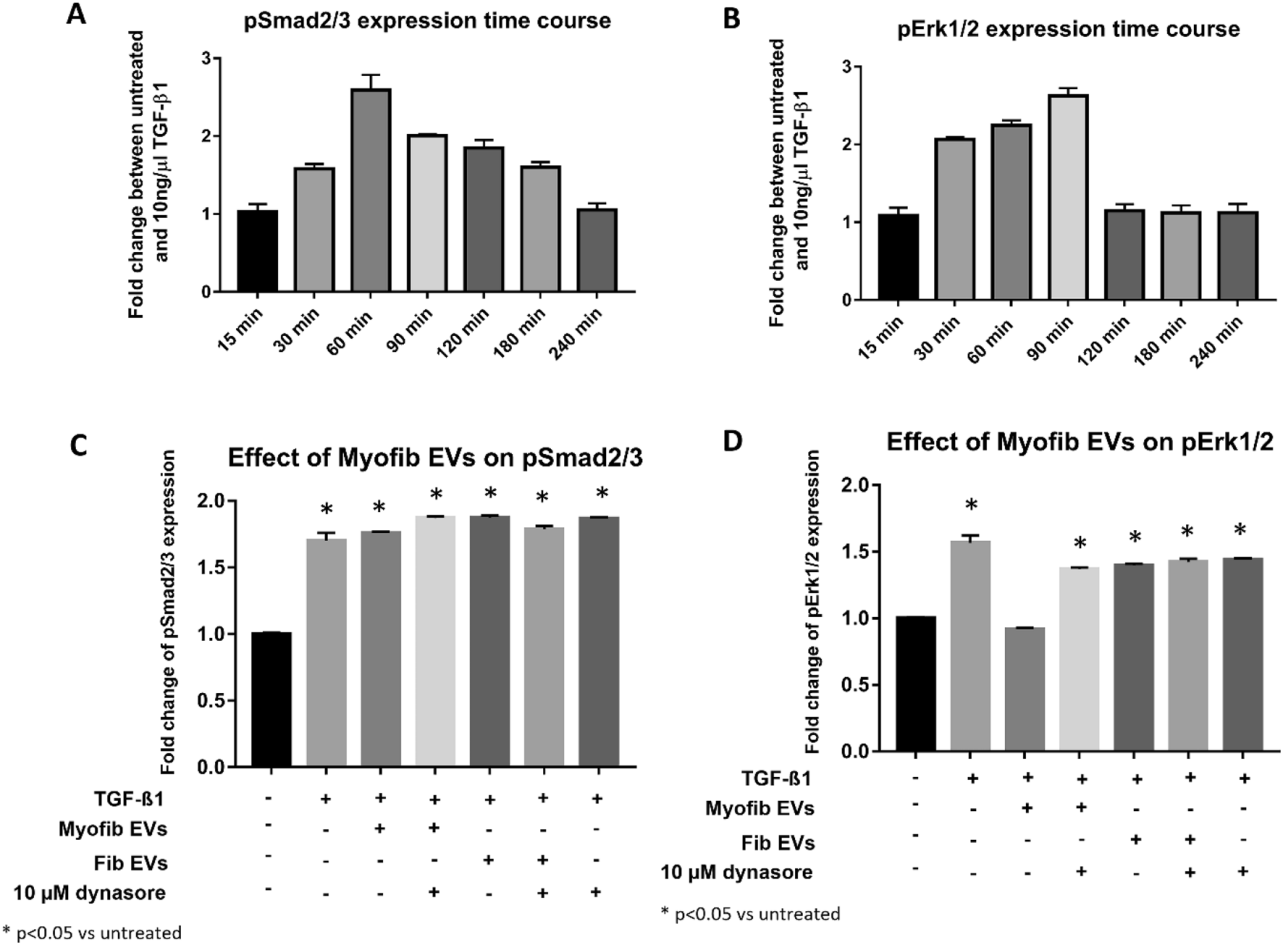
Effect of myofibroblast-derived EVs on TGF-β1 signalling. Downstream effectors of TGF-β1 were investigated (pSmad2/3, pErk1/2). Peak phosphorylation of effectors was assessed via time course of TGF-β1 treatment for pSmad2/3 (**A**) and pErk1/2 (**B**). Cells were incubated with TGF-β1 and/or fibroblast and myofibroblast EVs (6 × 10^8^ /mL) in presence or absence of 10 μM dynasore for 60 min (pSmad2/3) or 90 min (pErk1/2) based on the peak phosphorylation observed in (**A**) and (**B**). None of the vesicles affected Smad phosphorylation (**C**). Myofibroblast EVs prevented Erk1/2 phosphorylation which could be abrogated via dynasore (**D**). *p < 0.05 vs untreated cells. N = 3.

The phosphorylation of Smad2/3 peaked after 1 h (Fig. 7A), whilst Erk1/2 phosphorylation peaked after 90 min (Fig. 7B). These points were chosen for subsequent co-incubation of vesicles with TGF-β1 and/or 10 µM dynasore. Whilst myofibroblast-derived EVs did not affect phosphorylation levels of Smad2/3 (Fig. 7C), the phosphorylation of Erk1/2 was wholly abrogated by myofibroblast-derived EVs, which could again be prevented by addition of dynasore, further substantiating that vesicle uptake is necessary for the observed effect (Fig. 7D). Fibroblast-derived EVs did not affect either Smad2/3 or Erk1/2 phosphorylation (Fig. 7C and D).

### Investigating the protein cargo of myofibroblast-derived vesicles

**Figure 8:**
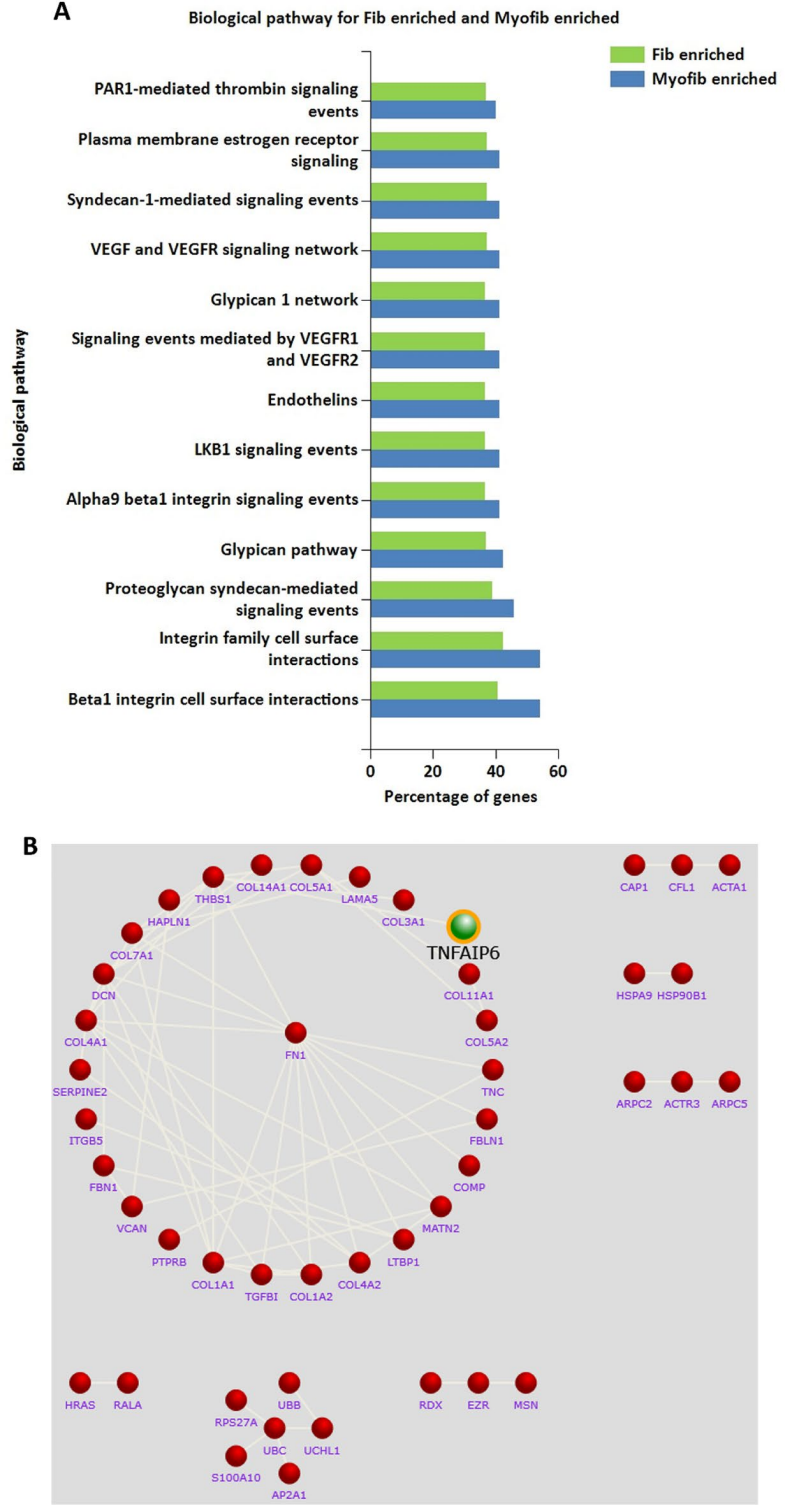
Proteomic analysis of EVs to decipher anti-fibrotic cargo. Proteins enriched in fibroblast and myofibroblast EVs were analysed to decipher anti-fibrotic cargo. (**A**) Observational datasets were generated by comparing the means of the replicates and clustering the ones in respective groups (higher in fibroblasts or in myofibroblasts) and subjected to biological process enrichment analysis. Graph displays percentage of genes enriched for the processes. (**B**) Interactome analysis performed using FunRich. Protein–protein interactions shown for myofibroblast-enriched proteins. Red nodes show selected proteins, interaction links in beige. Green node to highlight TSG-6.

The proteomics data of fibroblast-derived and myofibroblast-derived vesicles were compared in order to track down the potential effector proteins of their biological effect. The proteins were initially sorted by the means of the replicates which generated two protein sets that showed 376 proteins enriched specifically in the fibroblast-derived EVs and 107 in the myofibroblast-derived vesicles, generating two observational protein sets. The FunRich tool was used to compare the biological pathways of these two sets (Fig. 8A). Whilst these displayed no major differences, they did reveal a higher percentage of myofibroblast-derived vesicles being involved in integrin cell surface interactions. An interactome for myofibroblast-derived vesicles was generated (Fig. 8B) to understand known and predicted protein–protein interactions, which placed the myofibroblast protein fibronectin (FN1) at the centre. Of the nine proteins that were significantly upregulated when applying more stringent statistical analysis for upregulation, TNFAIP6 (TSG-6) was of particular interest (Table 1). It has been previously shown to act on Erk1/2, as well as prevent myofibroblast transformation^38,39^ and interactome analysis suggests that TSG-6 acts on thrombospondin, which has been suggested to regulate inflammation^40^.

**Table 1:** Statistical analysis of myofibroblast EV proteomic data was performed using multiple t-tests, and discovery was determined using the Two-stage linear step-up procedure of Benjamini, Krieger and Yekutieli, with Q = 5%.

Gene name	Fold-change	p value	q value
VCAN	3.2	0.000044544813081	0.003367587868942
KIAA1109	2.4	0.000219651471768	0.008302825632824
COL5A2	3.1	0.002332339191031	0.038082089185462
FRMPD1	2.8	0.002614326568253	0.038082089185462
TNFAIP6	7.3	0.002723211680956	0.038082089185462
TGFBI	13.7	0.003022388030592	0.038082089185462
THBS1	3.4	0.003809856350061	0.041033910226781
HAPLN1	1.9	0.004342212722411	0.041033910226781
COL7A1	3.3	0.005831064042339	0.048980937955647

Nine proteins were significantly upregulated compared to the Fib EV sample, with a q value (p value that was adjusted for false discovery rate of 5%) below 0.05.

## Discussion

### Characteristics of myofibroblast-derived EVs

Our results show that myofibroblast-derived EVs were greater in number but smaller and more elongated than the EVs produced by fibroblasts. Several studies have demonstrated increased EV production in animal models of fibrosis and in human samples from fibrotic diseases. For example, bronchoalveolar lavage fluid from both experimental bleomycin-induced mouse lung fibrosis models and from patients with idiopathic pulmonary fibrosis showed increased EVs, particularly exosomes^41^. In systemic sclerosis, it was reported that fibroblasts from fibrotic tissue produce more EVs in general and specifically more tetraspanin-positive EVs compared to fibroblasts from non-fibrotic tissue^42^. Similarly in the kidney, EVs produced by tubular cells have been shown to be increased in fibrotic conditions^43^. Furthermore, although TGF-β1 has been previously suggested to regulate EV biogenesis in other cell types^44^, as evidenced by altered mRNA and protein content in vesicles derived from TGFRB2 deficient cells^45,46^, the exact impact of TGF-β1 on the vesicle content and characteristics has been unclear. Our results show that TGF-β1 stimulation of myofibroblasts leads to an approximately sevenfold increase in EV numbers and that these were smaller and more elongated than the EVs produced by fibroblasts. This is concomitant and consistent with the observed significant increase in gene expression of cholesterol synthesis and de novo lipogenesis*,* which are essential in EV biogenesis.

TEM confirmed vesicle morphology and size distribution, whilst proteomic analysis confirmed marker expression of frequently used EV markers. We have demonstrated that myofibroblast-derived EVs express the tetraspanin marker CD9 and CD63, as well as 80 of the top 100 proteins frequently identified in exosomes (http://www.exocarta.org). Additionally, expression of TSG101 in absence of calnexin further supports vesicle status^24^.

### Prevention of myofibroblast transformation by myofibroblast-derived EVs

Having established that myofibroblast-derived EVs were significantly different in numbers and distinctive in size and shape compared to those derived from fibroblast, we investigated whether myofibroblast-derived EVs could affect the transformation of fibroblasts to myofibroblasts in response to TGF-β1. Previous studies have shown EVs from lung^41^, kidney^47^, liver^48^ and cardiac^49^ fibrosis to be pro-fibrotic (reviewed in Ref.^50^). Much of the research in this field has also focused on the tumorigenic effect of cancer-associated fibroblast (CAF) EVs in cancer recipient cells, by promoting epithelial-to-mesenchymal transition (EMT), invasion, metastasis, stemness, growth, chemoresistance, and metabolic reprogramming, as well as inhibiting apoptosis^51^. Further, EVs have been suggested to drive fibrosis by regulating a variety of signalling pathways such as Wnt/β-catenin, Notch, YAP/TAZ, PTEN, AKT, PPAR, HIF, NF-κB, CXCR/CXCL, and MAPK/ERK^50^. There is at least one study reporting that the incorporation of TGF-β1 and TGFBRIII into the surface of carcinoma-derived EVs can lead to myofibroblast transformation in stromal fibroblasts^44,52, 53^. However, we have ruled out the possibility that the myofibroblast EVs contain TGF-β1 incorporated on the outside of the vesicles, for example as a remnant of conditioning the media by adding TGF-β1, since the anti-fibrotic effect is clearly uptake-dependent, evidenced by the inhibition using dynasore. Crucially, additional TGF-β1 on the vesicle surface would cause the opposite of what was observed, increasing TGF-β1 signalling and causing further myofibroblast transformation rather than preventing it. In PD, it was shown that mouse pericyte-derived extracellular-vesicle mimetic nanovesicles could affect gene expression in human tunica albuginea derived fibroblasts^54^, but the vesicles produced by the fibroblasts themselves have not been studied. Our results show an anti-fibrotic effect of the myofibroblast-derived EVs, as they prevented the TGF-β1-dependent transformation of fibroblasts to myofibroblasts. Whilst this disagrees with a number of previous studies, it is interesting to note that EVs derived from stem cells have been suggested to have anti-fibrotic properties in models of lung^55^, renal^56^, and cardiac fibrosis^57^. For example, EVs obtained from bone marrow-derived mesenchymal stem cells have been shown to prevent or reverse bleomycin-induced pulmonary fibrosis in mice^55^ and suppress activation of pulmonary fibroblasts in vitro^58^. Most of the anti-fibrotic effect of stem cell-derived EVs have been attributed to the anti-fibrotic micro-RNA (miRNAs) cargo in the EVs^50^.

The discrepancy with some of the established literature may be due to differences in technical details and experimental designs. Only two studies have utilised a protocol similar to ours, i.e. isolating EVs from conditioned media obtained from activated fibroblast and testing their effect on non-activated fibroblasts^42,59^. The results from one of these agree with ours, whereas the other study disagrees. Although the study by Lacey and colleagues suggests that IL-1β-activated human lung fibroblasts produce EVs that can inhibit TGF-β1-induced myofibroblast transformation, they show anti-fibrotic effects of IL-1β are obtained on its own without controlling for potential incorporation of it into vesicle membrane after the 24 h incubation. Further, the authors did not test any other fractions that were co-isolated (soluble protein fractions) and, most surprisingly, the vesicles from untreated fibroblasts also showed an anti-fibrotic effect. The Nakamura paper suggests that SSc-derived fibroblasts produce vesicles that further promote myofibroblast transformation in normal skin fibroblasts, evidenced by the elevated mRNA expression for collagen I. Importantly, there are several limitations to that study. First, it lacks any characterisation of whether the myofibroblasts in their SSc group were fully transformed. Second, there are technical questions around the validity of the RT-qPCR results used to support the authors conclusions, as there is no information on how RNA quality was assessed, and limited information on how RT and PCR reactions were carried out, what controls were included, the choice of reference genes, as well as PCR efficiencies. Furthermore, the fold-changes in mRNA expression are very low. Importantly, whereas that study added EVs derived from a SSc patient to cells derived from a non-SSc patient, in our study the vesicles were put back onto the cells of the same patient.

Importantly, and again in contrast to the results reported for the carcinoma-derived EVs, the anti-fibrotic effect of our myofibroblast-derived EVs was uptake-dependent, as evidenced by the inhibition of this effect by dynasore and the absence of any effect with non-myofibroblast media. We hypothesise a negative feedback loop in the TA-derived fibroblasts, where TGF-β1-induced myofibroblasts release EVs that are taken up by target cells and prevent further myofibroblast transformation. This is similar to the observation that TGF-β1 can upregulate inhibitory Smads such as Smad6 and Smad7^60^. Therefore, we can conclude that although our results do not agree with the current understanding that considers EVs to be pro-fibrotic, PD-associated transformed fibroblasts contain a cargo with anti-fibrotic properties similar to those found in stem cells.

### Effect of myofibroblast-derived EVs on TGF-β1 signalling

Our data show that both canonical (Smad) and non-canonical (non-Smad) signalling pathways can be activated by TGF-β1 in our cells, evidenced by peak phosphorylation of pSmad2/3 after 60 min and pErk1/2 after 90 min. In the Smad pathways, receptor Smads, Smad2 and Smad3 are phosphorylated via an activated TGF-β1 receptor and subsequently accumulate in the nucleus to regulate gene transcription^61^. Here we show that the anti-fibrotic effect of our vesicles is independent of Smad2/3 phosphorylation, indicating that non-canonical signalling is more likely to be causing the inhibition. However, it is conceivable that the EV cargo affects other Smads such as co-Smad Smad4 which needs to form heterodimers with pSmad2/3 to enter the nucleus^62^, or the inhibitory Smad7^63^. Our data clearly demonstrate that Erk1/2 phosphorylation is prevented by the vesicle cargo (prevented in presence of a vesicle uptake inhibitor). Non-canonical TGF-β1 signalling can include various signalling pathways such as Erk MAPK pathway, Rho-like GTPase pathways or PI3K/AKT pathways^17^. Whilst p38/MAPK can also be activated via TGF-ß1, it has been shown that this alone is not sufficient to cause an EMT response^64^. Rapid phosphorylation of Erk in response to TGF-β1 has been described in fibroblasts^65^, which suggests that the response does not require protein translation and is therefore not indirect. In cardiac stem/progenitor cells, allogenic stem cell progenitor-derived EVs induced MAPK/pERK1/2 activation, so there is precedent of the signalling cascade being affected by EVs^66^. Additionally, pErk1/2 signalling could be affected using exercise-induced EVs in cardiomyocytes^67^. Melanoma stem cells have been shown to produce EVs containing miR-592 which acts upstream of pErk1/2 and activtates the signalling cascade, leading to pro-metastatic activity^68^. This allows us to speculate that miRNAs are potentially drivers for our observed effects as well but affecting upstream components of pErk to prevent rather than exacerbate the signalling response, such as let-7d^69^.

### Identifying a candidate for the biological effect

To investigate which protein could be the cause for the prevention of Erk phosphorylation, we analysed the proteomics data to see which proteins were enriched in fibroblasts and myofibroblasts respectively. Using these datasets, the biological process analysis did not reveal any major differences that could have explained the anti-fibrotic action only observed in myofibroblast vesicles. In contrast, Interactome analysis combined with more stringent statistical analysis identified a group of nine proteins that were significantly upregulated, one of which was TSG-6 (7.3-fold-change, p < 0.05, q < 0.05).

Whilst a literature search on all nine upregulated proteins was conducted to investigate their involvement on ERK signalling and fibrosis, there is a fair body of evidence to hypothesize that the observed anti-fibrotic effect could be mediated through TSG-6, as outlined below.

TSG-6 plays a crucial role in counteracting TGF-ß-induced fibrosis by regulating extracellular matrix turnover, attenuating inflammation, and inhibiting TGF-ß signalling. It can bind to and promote the clearance of hyaluronan, a major component of the extracellular matrix that orchestrates TGF-β1-dependent maintenance of the myofibroblast phenotype^70^, thus helping to prevent hyaluronan accumulation in fibrotic tissues^71^. Furthermore, TSG-6 can modulate the immune response through a variety of means. It exerts an anti-inflammatory function by counteracting the transcription of MMP-1 and MMP-3 and the activation of MMP-1^72^, can induce the expression of the anti-inflammatory cytokine IL-10^73^ and downregulate the expression of the pro-inflammatory cytokine TNF-α^74^. Additionally, TSG-6 can help to attenuate the fibrotic process by inhibiting the activation and migration of neutrophils and macrophages^75,76^, which are involved in the inflammatory response associated with fibrosis. Finally, TSG-6 inhibits the activation of TGF-beta receptor I (TGFβRI) and downstream Smad signalling pathways^77^, thus blocking the activation of TGF-beta signalling. The interactome shows TSG-6 connects to thrombospondin, which has been previously described to be an important mediator of inflammation^40^, whilst upregulated TSG-6 expression in adipose tissue derived stem cells has been suggested to prevent inflammation^78^. TSG-6 has also been suggested as a potential treatment strategy for renal inflammation^79^. Additionally, TSG-6 has been described to prevent phosphorylation of Erk and p38^38^. Further, TSG-6 has been attributed with prevention of myofibroblast transformation and reduction of ECM production when secreted by mesenchymal stem cells or contained within mesenchymal stem cell-derived EVs^39^. In mouse models of full-thickness wounds, it could be shown that TSG-6 reduced tissue fibrosis and myofibroblast transformation^80^.

Several studies have demonstrated the presence of TSG-6 in EVs. For example, EVs derived from mesenchymal stem cells (MSCs) have been shown to carry TSG-6 on their surface or encapsulated within their cargo^81,82^. These TSG-6-containing EVs are implicated in various therapeutic effects and have been shown to reduce inflammation, promote tissue regeneration, and modulate immune responses^83,84^.

In this study, we have demonstrated that myofibroblast-derived EVs contain an anti-fibrotic protein called TSG-6 which is likely to exert negative feedback on the myofibroblast transformation. Such negative feedback would suggest that PD should have been a self-limiting disease, i.e. myofibroblasts should be able to stop transformation of new myofibroblasts therefore the fibrotic tissue should clear itself. However, it is established that PD is a chronic and progressive disease, it is not self-limiting. This implies that this negative feedback system may not be efficient or sufficient to control the progression of fibrosis in PD. One possible explanation for this could be that TGS-6 might be downregulated as reported in cancer associated fibrosis^85^ which could lead to less effective negative feedback hence more progressive fibrosis. It can therefore be hypothesised that up regulation of this negative feedback system may be an effective way of treating PD. Several studies have actually reported that exogenous administration of TSG-6^80,86^ have anti-fibrotic effects. Further investigation into these EVs and TSG-6 may lead to discovery of novel medicines for not only PD but also for other fibrotic diseases and cancer metastasis^85^.

### Limitations

One of the limitations of this study is that we only investigated the vesicles produced by fibroblasts derived from patients with PD. Having access to a multitude of primary human fibroblasts from various tissue sources, it would be interesting to see if this effect could be replicated using fibroblasts from different sources, e.g., if skin myofibroblast EVs can prevent myofibroblast transformation of skin fibroblasts. Further, it would be interesting to see if the effect could be seen across tissues, e.g., can the skin or PD vesicles inhibit myofibroblast transformation of cardiac or lung fibroblasts. In terms of fibroblast heterogeneity, this would have potential to a significant contribution to the fibrosis field, as we previously reported that exposure to the same FDA-approved drugs can lead to different levels of myofibroblast transformation or prevention thereof in various tissues, i.e. drugs that show significant inhibition in one tissue type show no inhibition in the other^87,88^. In addition, in the current study, we only tested one time point of adding the myofibroblast vesicles (co-incubated with TGF-β1). We have previously established that myofibroblast transformation has a point-of-no-return after 36 h of TGF-β1 treatment. Therefore, testing whether the vesicles can prevent or reverse the myofibroblast transformation process any further after the initial TGF-β1 dosing would be interesting. Further validation is needed to confirm that TSG-6 is responsible for the anti-fibrotic effect observed.

## Conclusion

In conclusion, our study is the first description of EVs isolated from fibroblasts and myofibroblasts derived from tunica albuginea of patients with Peyronie’s disease. Further, this is the first study to suggest that PD cells that were treated with TGF-β1 (myofibroblasts) produce vesicles with anti-fibrotic effect that can abrogate TGF-β1 signalling. We hypothesize that this effect might be linked to the enrichment of the anti-fibrotic factor TSG-6. Our results point towards a strategy that has potential to be exploited for therapeutic use and provides a new avenue of research for fibroblast biology. Further research is required to understand the mechanism of action of TSG-6 on fibrotic pathways.

## Material and methods

### Ethics statement

Ethical approval was obtained by independent research ethics committees (NHS Research Ethics Committee East of England [12-EE-0170] and North of Scotland [15-NS-0051]). Patients that were included were between 18 and 75 years old and able to understand the patient information sheet. Informed consent was obtained from all patients. All methods were carried out in accordance with relevant guidelines.

## Isolation of fibroblasts

Human primary fibroblasts were isolated from TA samples from patients undergoing corrective surgery for PD as previously described^88–91^. Tissue samples were transferred to the labs from the hospital in ice cold DMEM F-12 with 10% foetal calf serum (FCS) and 1% penicillin/streptomycin in less than 3 h. Once the tissue arrived in the lab, it was dissected and corpus cavernosum was removed. The tissue fragments (5 mm × 5 mm) were anchored to the bottom of a 6-well plate and submerged in DMEM F-12 with 10% FCS and 1% penicillin/streptomycin and placed in the 37 °C, 5% CO_2_ incubator. Cell outgrowth was observed after 7 days after which the tissue fragments were removed. Cells were grown to confluency and expanded in T75 flasks (Thermo Fisher Scientific). The fibroblasts were characterised as previously described and cell identity was confirmed by expression of vimentin in the absence of expression of desmin^88–90^.

Rhabdomysarcoma (RD) cells were purchased from Sigma Aldrich and supplied by European Collection of Authenticated Cell Cultures (ECACC) operated by Public Health England. These cells were cultured in DMEM F-12 with 10% FCS and 1% penicillin/streptomycin and placed in the 37 °C, 5% CO_2_ incubator for cell culture.

### Induction of myofibroblast transformation

To induce the phenotypic switch of fibroblasts to myofibroblasts, we treated primary human fibroblasts with 10 ng/mL TGF-β1. This concentration was previously determined to induce full myofibroblast transformation after 72 h^89–91^. Myofibroblasts could be distinguished visually (phase contrast microscopy) and by quantifying expression of the myofibroblast marker α-SMA using immunocytochemistry and in cell ELISA, as previously described^88–91^.

### Isolation of extracellular vesicles from conditioned media

Cells were grown to near confluence in T75 flasks in DMEM F-12 with 10% FCS and 1% penicillin/streptomycin. The media was then changed to serum-free KOSR media (10% Knockout Serum Replacement (KOSR; Gibco) in DMEM F-12 with 1% penicillin/streptomycin) overnight before addition of TGF-β1. KOSR is a defined serum replacement^32^ and serum-free KOSR media was used to avoid EV contamination from FCS. After 24 h of TGF-β1-incubation, cells were washed with PBS and fresh serum-free KOSR media was added. After 48 h, the media was collected and subjected to centrifugation (10 min at 4000 *g* at 4 °C) to pellet cell debris. The supernatant was concentrated using 100 kDA filters (Amicon) using a centrifuge at 4000 *g* for 20 min at 4 °C. Concentrated media (0.5 mL) was then subjected to size-exclusion chromatography using 70 nm qEV columns (Izon) to isolate EVs and protein fractions, according to the manufacturer’s instructions. Fractions were collected as 1.5 mL fractions after the column void volume was discarded. The three fractions were EV fraction, protein fraction 1 (early eluting proteins) and protein fraction 2 (late eluting proteins). Controls included KOSR media only and conditioned media obtained from human RD rhabdomyosarcoma (RD) cells grown in serum-free KOSR media.

## Assessment of cell viability

Before collection of conditioned media, cell numbers and viability were assessed to ensure these would not affect EV results. Images were taken using an Olympus microscope with a Leica DFC300 camera. Cells were counted using a Sceptre Cell Counter (Merck-Millipore) and viability was assessed using trypan blue.

### Immunocytochemistry

To visualise α-SMA expression in cells, immunocytochemistry (ICC) was utilised. 50,000 cells per well were seeded onto sterile cover slips in a 6-well plate (NUNC, Fisher Scientific, UK) and incubated overnight at 37 °C, 5% CO_2_ in a humidified atmosphere. The next day, the cells were treated with blank media or media containing 10 ng/mL TGF-β1 to induce myofibroblast transformation. After 72 h incubation at 37 °C, 5% CO_2_ in a humidified atmosphere, the cells were fixed using ice-cold methanol and unspecific binding was blocked using 10% donkey serum (Millipore) in PBS for 1 h. Primary antibody against α-SMA (raised in mouse) was added at 1:1,000 (Sigma) for 2 h. This was followed by three washes with PBS before incubation with the secondary antibody (1:1,000, Abcam; raised in donkey). After 2 h, cover slips were washed and mounted using VECTASHIELD^®^ mounting medium with DAPI (Vector Laboratories, UK). Images were taken using an Olympus IX71 fluorescent microscope and a Leica DFC3000 G camera and LASX software (Leica, Germany).

### Negative staining and transmission electron microscopy (TEM)

To prepare samples for TEM, they were negatively stained. Briefly, 300-mesh copper carbon-filmed grids were glow discharged with plasma to improve hydrophilicity. Then, 3.5 µl of the sample was pipetted onto the grid, and allowed to absorb onto the film for 20 min. The grids were then fixed with 2.5% glutaraldehyde for 1 min, and washed 3 × in distilled water. 3% uranyl acetate solution was added onto the side of the grid with the sample, and allowed to incubate for 1 min. The solution was blotted off to a filter paper and grids allowed to dry overnight.

The samples were then imaged using a Jeol-1400 TEM (Japan) at accelerating voltage of 80 kV and images were taken at various magnifications (100 nm, 200 nm, 400 nm).

### Proteomics analysis

EVs were concentrated with size exclusion chromatography using qEV Concentration Kit (Izon) according to the manufacturer’s instructions. The 1.5 mL sample obtained from qEV size exclusion column was concentrated using Izon’s sEV Concentration Kit (Izon) by mixing the sample with 100 μl of supplied beads and incubated for 1 h on a rotary shaker. Mixture was centrifuged at 16,000 g for 10 min to pellet EVs after which the pellet was resuspended and lysed in RIPA buffer (Sigma). The immunoprecipitated proteins were alkylated, reduced and digested overnight at 37 °C using dithiothreitol, iodoacetamide and Promega Gold Trypsin Lys-C. The reaction was ceased using trifluoracetic acid then desalting and cleaning was conducted using Thermo Hypersep C^18^ tips. Reconstituted samples were injected into a Sciex ZenoTOF 7600 via a waters M-Class HPLC and Kinetex^®^ 2.6 µm XB-C^18^ column. Data were acquired in zenoSWATH Data Independent Acquisition (DIA) mode over a 17 min HPLC run on a 150 × 0.3 mm Phenomenex Kinetex XB-C18 2.6 um column at 10 µl /min (11 min gradient, 3–40% acetonitrile/0.1% formic acid with 80% wash phase prior to re-equilibration) optimised for complex samples. DIA-NN software was used to search the data against Uniprot human proteome and common lab contaminants.

### Western blot

EVs were concentrated with size exclusion chromatography using qEV Concentration Kit (Izon) according to the manufacturer’s instructions. The 1.5 mL sample obtained from qEV size exclusion column was concentrated using Izon’s sEV Concentration Kit (Izon) by mixing the sample with 100 μl of supplied beads and incubated for 1 h on a rotary shaker. The mixture was centrifuged at 16,000 g for 10 min to pellet EVs after which the pellet was resuspended and lysed in RIPA buffer (Sigma). The samples were resuspended by heating samples to 95 °C for 5 min and then mixed with 4 × loading buffer (Li-Cor) under non-reducing conditions. Samples were loaded onto Any kD Mini-PROTEAN^®^ TGX Precast Protein Gels (Bio-Rad) along with a protein ladder (Bio-Rad). The samples were transferred to methanol activated PVDF membranes using wet blotting (350 mV for 1 h). Membranes were left to dry for 1 h and then stained using Revert 700 stain (Li-Cor) following the manufacturer’s instructions to ensure optimum transfer of proteins by visualising all proteins. Membranes were visualised with infrared plate reader (Odyssey CLx, Li-Cor) using 700 nm channel and then blocked using Intercept buffer (Li-Cor). Membranes were cut before incubation with primary antibodies raised against human CD9, CD63, Calnexin and TSG-101 (Abcam, 1:1000 in Intercept buffer with 0.2% Tween-20) overnight at 4 °C. Membranes were washed 4 times using TBS with 0.1% Tween and then incubated with secondary antibody against rabbit (800 nm, 1:10,000 in Intercept buffer with 0.2% Tween-20 and 0.01% SDS; Li-Cor) for 1 h at room temperature in the dark. Membranes were washed 4 times with TBS with 0.1% Tween-20 and scanned with infrared plate reader (Odyssey CLx, Li-Cor) using both 700 nm and 800 nm wavelengths.

### Particle analysis using tunable resistive pulse sensing

To characterise EVs and to determine to determine size, concentration and shape of the vesicles, a particle analyser (qNano Gold; Izon) was utilised. The qNano Gold uses Tunable Resistive Pulse Sensing (TRPS) technology to analyse particles using Izon Control Suite 3.4 software. EVs were filtered and diluted in PBS (1:5–1:100 depending on measurement). Prior to analysis, all samples were vortexed and then loaded into the upper fluid chamber of the analyser. Flexible polyurethane membrane nanopores (NP150 Nanopore, Izon) were used for the measurements which cover the particle sizes from 70 to 420 nm. Standard particles (CPC100; Izon) were used to calibrate the measurements. Calibrations were alternated with sample measurements if pressure was changed for optimal sample flow, thereby guaranteeing reliable results. The system was washed with distilled water between sample runs to ensure only relevant sample was detected and any residual vesicles were avoided. All replicate samples were analysed using the same Nanopore.

### In-cell ELISA

To quantify the expression of the myofibroblast marker alpha-smooth muscle actin, as well as the phosphorylation status of Smad2/3 and Erk1/2, we used the In-Cell ELISA (ICE) technique, as previously described and validated^88–90^. Depending on experimental design, different cell numbers were seeded onto 96-well optical flat-bottom black microplates (Nunc, Rochester, New York). For quantification α-SMA, 5,000 cell/well were seeded followed by co-incubation with various concentrations of vesicles and protein fractions in presence of 5 ng/mL TGF-β1 for 72 h. As fibroblasts produced fewer vesicles than myofibroblasts, the concentrations were adjusted so that they were equal. We tested the following vesicle concentrations: 6 × 10^8^/mL, 2 × 10^8^/mL, and 0.6 × 10^8^/mL. Protein fractions were diluted accordingly. To quantify the phosphorylation levels of Smad2/3 and Erk1/2, 10,000 cells/well were seeded, followed by a length of TGF-β1 incubation (15–240 min) that corresponds to the peak phosphorylation level of the protein in the absence or presence of myofibroblast-derived EVs with or without 10 μM of dynasore (Sigma Aldrich). Following the incubation period at 37 °C and 5% CO_2_, cells were fixed using 4% paraformaldehyde for 20 min at room temperature. Cells were permeabilised by 3 washes using PBS with 0.1% Triton-X, which was followed by a 90 min incubation with 10% donkey serum in PBS with 0.1% Triton-X to block unspecific binding. Primary antibodies were added to the cells for a-SMA (1:3,000; Sigma-Aldrich), pSmad2/3 (1:250; Abcam) or pErk1/2 (1:250; Abcam). Following the 2 h incubation, cells were washed 3 × using PBS with 0.1% Tween-20. Secondary antibody (1:500; Li-Cor IRDye 800) and nuclear stain (1:1000; DRAQ5) were added and cells were incubated for 1 h in the dark. This was followed by three washes using PBS with 0.1% Tween-20, after which the plates were scanned using an infrared imaging system (Odyssey CLx imager; LI-COR, UK) at both 700 nm and 800 nm wavelengths. Protein expression data were normalised to nuclear staining and expressed as 800/700 ratio using Microsoft Excel.

### Biological process, pathways analysis, and protein–protein-interaction

Enrichment analysis comparing the multiple datasets obtained by proteomics was performed using FunRich software (v3.1.3; funrich.org), which allowed to investigate human databases (FunRich database) and Vesiclepedia database to identify enriched terms of biological processes and pathways, as well as protein–protein interaction network analysis.

### Statistical analysis

The number of samples (i.e., patients) was calculated using G*Power Software (version 3.1.99.7)^92^ and was based on effect size of 5.43, alpha error probability of 0.5 and 95% power. For statistical analysis between multiple groups, one-way analysis of variance (ANOVA) was used unless otherwise stated. Data expressed as mean ± SD. P less than 0.05 was considered statistically significant. For statistical analysis of myofibroblast EV proteomic data, multiple t-tests were performed, and discovery was determined using the Two-stage linear step-up procedure of Benjamini, Krieger and Yekutieli^93^ with q values (p value that was adjusted for false discovery rate of 5%) of below 0.05 considered significant, N = 4. Data were analysed using GraphPad Prism 7.0.

### Supplementary Information

Supplementary Information.

## Data Availability

Data will be made available upon reasonable request. Please contact corresponding author Prof Selim Cellek (selim.cellek@aru.ac.uk).
