# Supplementary material for: TGF-β1 induces formation of TSG-6-enriched extracellular vesicles in fibroblasts which can prevent myofibroblast transformation by modulating Erk1/2 phosphorylation
Source: Sci Rep. 2024 May 29;14:12389. doi: 10.1038/s41598-024-62123-x (PMC11136978; doi:10.1038/s41598-024-62123-x)
Supplement: Supplementary file 1 — Supplementary Information. [file 41598_2024_62123_MOESM1_ESM.pdf]

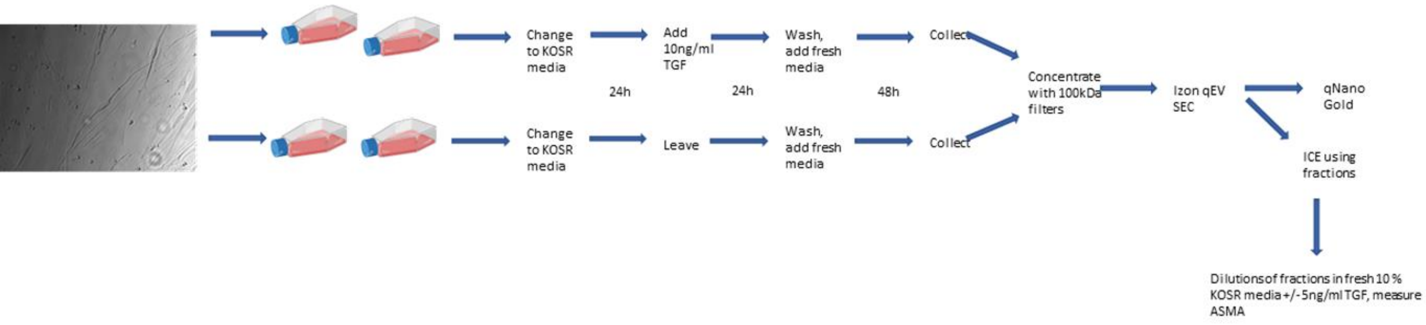

Supplementary Figure 1: Summary of vesicle isolation approach. Graphical summary of our approach to isolate and analyse EVs from primary human fibroblasts and myofibroblasts.

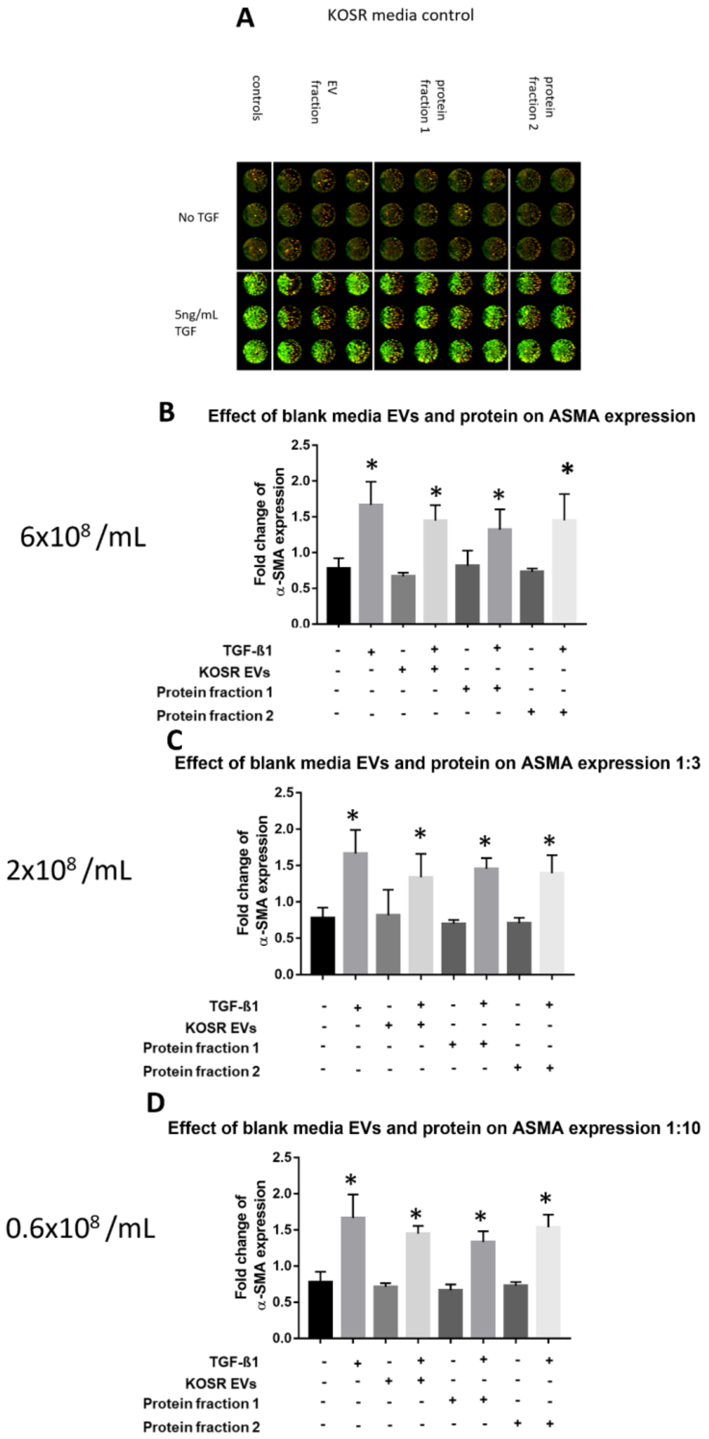

Supplementary Figure 2: Effect of KOSR media vesicles and soluble protein fractions on myofibroblast transformation. Fibroblasts were treated with various concentrations of KOSR derived EVs or soluble protein fractions in presence or absence of 5ng/mL TGF-β1 for 72h after which myofibroblast transformation was quantified using ICE for a-SMA. A: Odyssey image output showing plate layout and results with red indicating nuclear staining and green indicating a-SMA expression. B Quantification of highest concentration of EVs and soluble protein +/- TGF-β1. No effect shown for any EV or protein fraction. C Quantification of middle concentration of EVs and soluble protein +/- TGF-β1. No effect shown for any EV or protein fraction. D Quantification of lowest concentration of EVs and soluble protein +/- TGF-β1. No effect shown for any EV or protein fraction. *p<0.05 vs untreated cells. N=3

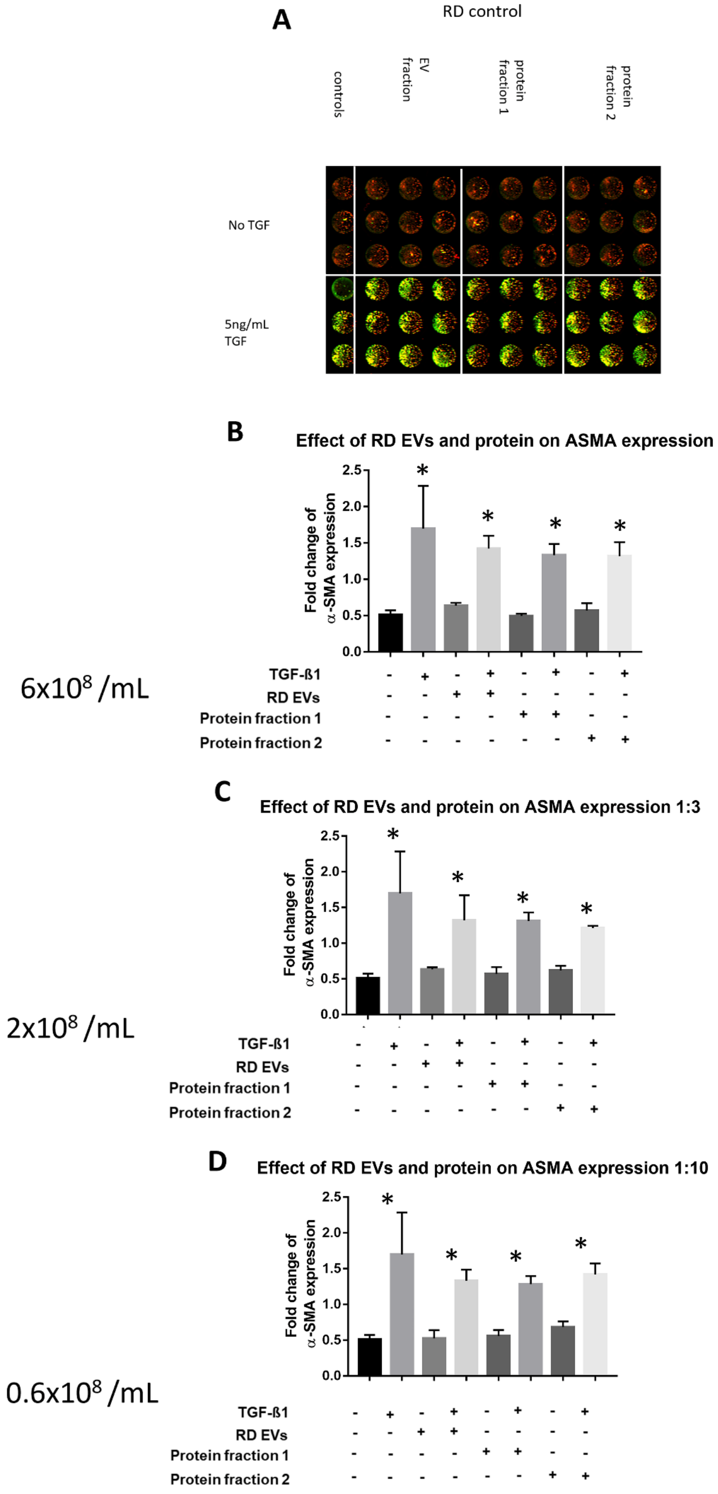

Supplementary Figure 3: Effect of rhabdomyosarcoma-derived vesicles and soluble protein fractions on myofibroblast transformation. Fibroblasts were treated with various concentrations of RD- derived EVs or soluble protein fractions in presence or absence of 5ng/mL TGF-β1 for 72h after which myofibroblast transformation was quantified using ICE for a-SMA. A: Odyssey image output showing plate layout and results with red indicating nuclear staining and green indicating a-SMA expression. B Quantification of highest concentration of EVs and soluble protein +/- TGF-β1. No effect shown for any EV or protein fraction. C Quantification of middle concentration of EVs and soluble protein +/- TGF-β1. No effect shown for any EV or protein fraction. D Quantification of lowest concentration of EVs and soluble protein +/- TGF-β1. No effect shown for any EV or protein fraction. *$p<0.05$ vs untreated cells. N=3

Supplementary Western blot details

A Revert 700 total protein stain

B CD9

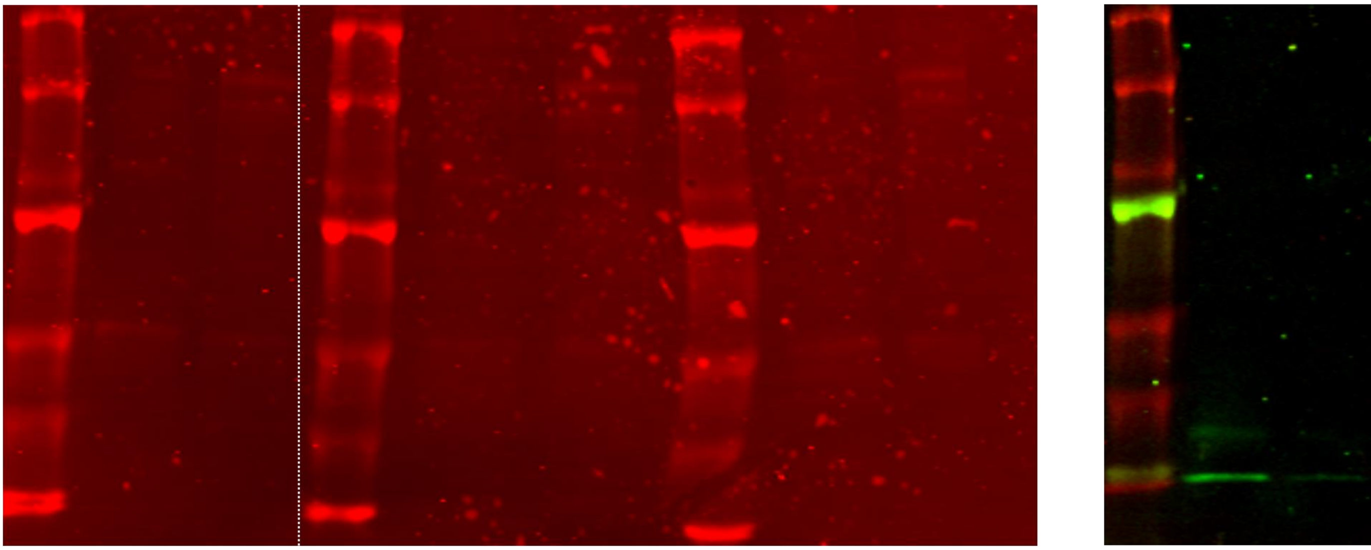

Membrane was stained using Li-Cor Revert 700 total protein stain and imaged using Odyssey plate reader at 700nm with red bands indicating protein being present (A). Membrane was then cut (dotted line) before incubation with primary antibody and subsequently with secondary antibody. Membrane was scanned at 700nm and 800nm, with green bands indicating specific staining of protein of interest (B).

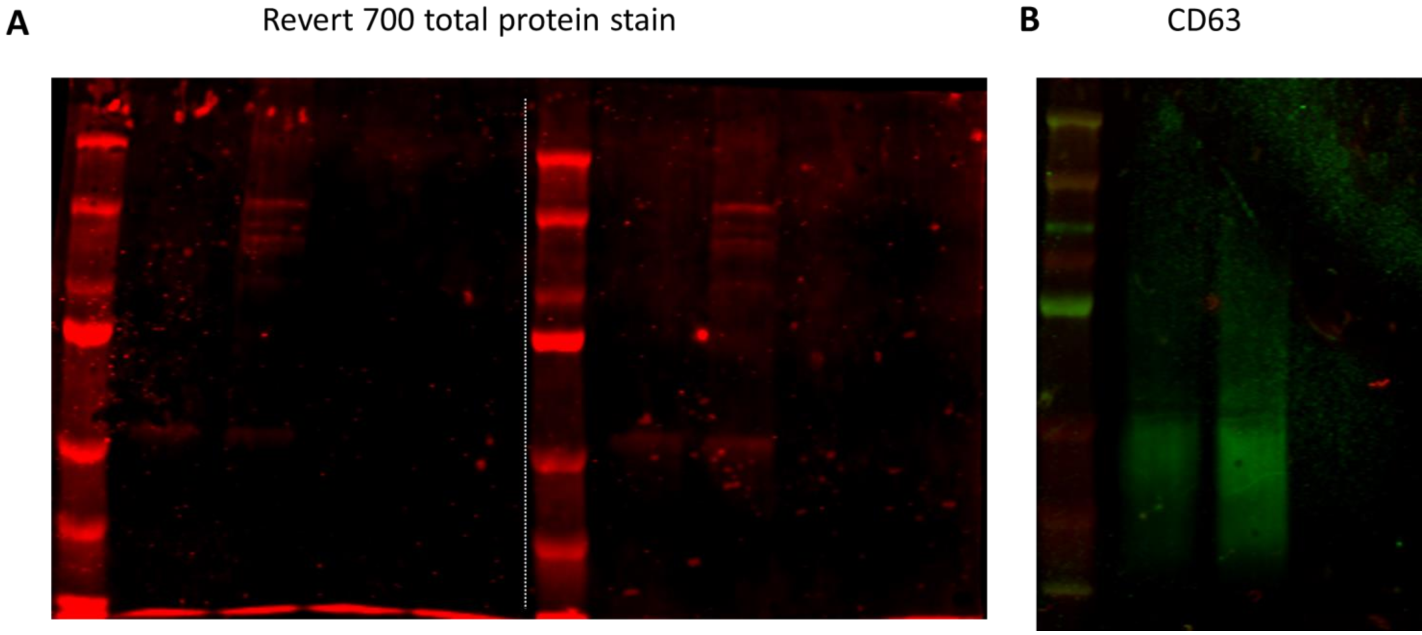

Membrane was stained using Li-Cor Revert 700 total protein stain and imaged using Odyssey plate reader at 700nm with red bands indicating protein being present (A). Membrane was then cut (dotted line) before incubation with primary antibody and subsequently with secondary antibody. Membrane was scanned at 700nm and 800nm, with green bands indicating specific staining of protein of interest (B).

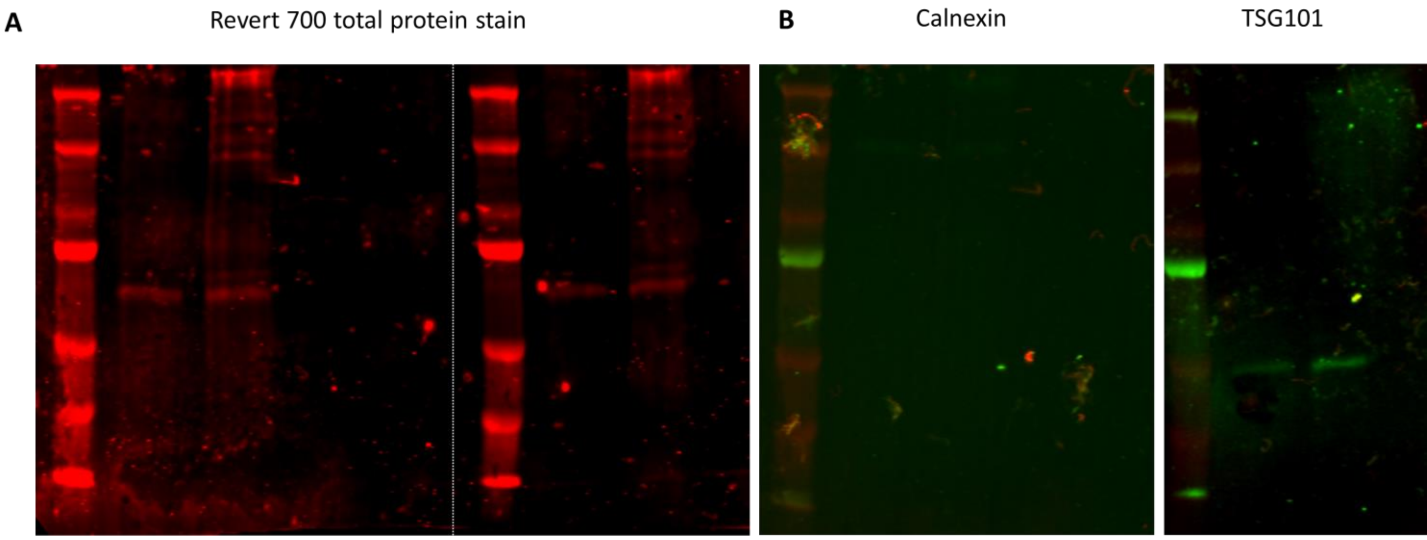

Membrane was stained using Li-Cor Revert 700 total protein stain and imaged using Odyssey plate reader at 700nm with red bands indicating protein being present (A). Membrane was then cut (dotted line) before incubation with primary antibody and subsequently with secondary antibody. Membrane was scanned at 700nm and 800nm, with green bands indicating specific staining of protein of interest (B).
